# Effects of Age and Environment on Adaptive Immune Responses to *Mycobacterium avium* subsp. *paratuberculosis* (MAP) Vaccination in Dairy Goats in Relation to Paratuberculosis Control Strategies

**DOI:** 10.3390/vetsci6030062

**Published:** 2019-07-01

**Authors:** Ad Koets, Lars Ravesloot, Robin Ruuls, Annemieke Dinkla, Susanne Eisenberg, Karianne Lievaart-Peterson

**Affiliations:** 1 Department of Bacteriology and Epidemiology, Wageningen Bioveterinary Research, 8200 AB Lelystad, The Netherlands; 2 Department of Farm Animal Health, Faculty of Veterinary Medicine, Utrecht University, 3584 CL Utrecht, The Netherlands; 3 GD Animal Health, 7418 EZ Deventer, the Netherlands

**Keywords:** paratuberculosis, vaccination, immunity, mycobacterium, diagnostics

## Abstract

Paratuberculosis infection is caused by *Mycobacterium avium subsp. paratuberculosis* (MAP). In the Netherlands, 75% herd level prevalence of caprine paratuberculosis has been estimated, and vaccination is the principal control strategy applied. Most goat dairy farms with endemic paratuberculosis systematically vaccinate goat kids in the first months of life with a commercially available whole cell MAP vaccine. We hypothesized that the development of adaptive immune responses in goats vaccinated at young age depends on the environment they are raised in, and this has implications for the application of immune diagnostic tests in vaccinated dairy goats. We evaluated the early immune response to vaccination in young goat kids sourced from a MAP unsuspected non-vaccinated herd and raised in a MAP-free environment. Subsequently we compared these with responses observed in birth year and vaccination matched adult goats raised on farms with endemic paratuberculosis. Results indicated that initial adaptive immune responses to vaccination are limited in a MAP-free environment. In addition, adult antibody positive vaccinated goats raised in a MAP endemic environment are less likely to be IS900 PCR-positive as compared to antibody negative herd mates. We conclude that test-and-cull strategies in a vaccinated herd are currently not feasible using available immune diagnostic tests.

## 1. Introduction

Paratuberculosis infection in ruminants including goats is caused by oral uptake of *Mycobacterium avium* subsp. *paratuberculosis* (MAP) from MAP-positive feces contaminated environmental sources [1]. MAP causes granulomatous lesions in the distal part of the ileum in domestic and wild ruminants. In goats, ileal lesions limit sufficient nutrient uptake and therefore goats suffering from clinical Johne’s disease predominantly show weight loss, skin peeling and a rough hair coat while diarrhea is rarely seen [1,2]. In the Netherlands, prevalence of MAP infection in dairy goats is not precisely known but it is estimated that over 75% of commercial Dutch dairy goat operations have endemic paratuberculosis based on clinical and routine (patho)diagnostic observations. Schuiling and Groeneveld [3] showed that Johne’s disease in combination with caprine arthritis encephalitis, had the highest economic impact on endemically infected farms in the Netherlands.

The main three strategies to control paratuberculosis on infected goat farms are management measures to reduce MAP transmission, test-and-cull-strategies to reduce sources of infection, and vaccination to decrease the susceptibility of the young stock [1,4]. Although these classical control strategies are able to reduce the rate of infection considerably, eradication of MAP has been shown to be difficult and additional approaches to address control of paratuberculosis are needed [1].

Management measures to reduce MAP transmission focus mainly on avoiding contact of young, susceptible stock with infected animals and their feces, such as separation of kids from dams immediately after birth. For test-and-cull strategies to reduce the sources of infection, ELISA tests are most commonly used because of their simplicity and low cost. In cattle, absorbed ELISA tests are considered to be highly specific, but of low sensitivity [1,5]. This is because the antibody response, as measured by commercial absorbed ELISA in bovine and caprine milk or serum, is associated with later and progressive stages of the infection and disease. Moreover, while the infection progresses the antibody mediated immune response may show temporal variation, which when close to the test cut-off may lead to intermittent positive results on repeated measurements [6]. When the animal progresses to advanced clinical stages of the disease, anergy may lead to false negative results. Antibody detection by ELISA, even in animals in advanced stages of infection, may, therefore, be unreliable when used as a single time point test. Repeated testing of goats over a longer time period can be applied to increase the probability of antibody detection in infectious animals and to reduce the sources of infection [7].

Vaccination is the principal control strategy for caprine paratuberculosis in the Netherlands. It is estimated that over 75% of farms with endemic paratuberculosis infection systematically vaccinate their goat kids in the first month of life against paratuberculosis with a commercially available whole cell vaccine. Bastida and Juste have summarized results of vaccination experiments and showed that MAP vaccination performed well in reducing production, epidemiological, and pathogenetic effects in cows, sheep and goats but does not prevent infection [4]. 

As has been reviewed in a number of aforementioned papers there is a paucity in the veterinary literature regarding the immunology of vaccination in goats despite the fact that vaccination is widely used on a global scale to control small ruminant paratuberculosis. Some characteristics of the vaccine, including the fact that vaccination does not prevent infection and MAP shedding can still occur despite efficient control of clinical symptoms, are poorly understood. In addition, several questions remain as to the possibility of performing immune diagnostics on vaccinated goats to differentiate the vaccinated non-infected from vaccinated infected individuals. 

Vaccination against paratuberculosis has been widely used on sheep and goat farms and a meta-analysis on efficacy shows that overall, vaccination is beneficial in control of paratuberculosis in small ruminants [4]. In Norway, a vaccination program was initiated in 1967 after several years of unsuccessful efforts to eradicate paratuberculosis in goats. The efficacy of the vaccine was judged mainly by postmortem examination of vaccinated and unvaccinated goats in the period 1967 to 1982. These results showed that MAP vaccination offers a high reduction of lesions [8]. In Australia, MAP vaccination of Merino sheep flocks was usually effective in reducing prevalence of shedding, but the response to vaccination in the different flocks was variable and shedding of MAP persisted in three out of eight flocks followed longitudinally over a decade [9]. 

A limited number of controlled studies on immune responses following MAP bacterin vaccination have been published [10,11,12]. Valheim et al. [10] used goat kids from MAP-vaccinated dams from farms on which no clinical paratuberculosis was observed. Results indicated that following vaccination at 3 weeks of age a peak in PPD specific IFN-γ response was observed one month later at 7 weeks of age followed by a rapid decline in the following 2 weeks. This was also reflected in the frequency of CD25 positive T cells in the vaccination site draining lymph node. MAP-specific antibody titers also increased between 3 and 7 weeks but leveled off at 9 weeks despite the active granulomatous lesions induced by the vaccine. Storset et al. [11] evaluated IFN gamma responses in goats from farms with or without MAP vaccination and different infection status. They reported that animals from vaccinated herds with paratuberculosis showed higher IFN-γ responses as compared to animals from the vaccinated only herds. In both these groups the responses were correlated to age with higher responses in younger animals. Corpa et al. [12] studied the effect of age at which goat kids were vaccinated and observed that between vaccinating at 15 days of age and 5 months of age there was no significant difference although IFN-γ responses tended to disappear earlier in animals vaccinated at 15 days old. The antibody response observed by Corpa et al. was always higher and more persistent in animals vaccinated at 5 months as compared to 15 days of age at vaccination, possibly related to maturity of the immune system at the different ages [12].

We hypothesized that the development of adaptive immune responses in goats vaccinated at young age is also dependent on the environment they are raised in. Recent data obtained in mice highlighted the effects of environment on the basal immune state and response to infection [13]. A secondary question was whether this also has implications or opportunities for immune diagnostic tests in vaccinated populations of dairy goats. The aim of the current study was therefore to first evaluate the early immune response to vaccination in young goat kids sourced from a MAP unsuspected non-vaccinated herd and raised in a MAP-free environment. Subsequently we compared these immune responses with the immune responses observed in adult goats which were similarly vaccinated as kids but raised on farms with documented endemic paratuberculosis.

## 2. Materials and Methods 

### 2.1. Environmental Sampling 

A total of 20 commercial dairy goat farms with a documented history of paratuberculosis located across the Netherlands volunteered to participate in the environmental sampling in the summer of 2012. All these farms housed their young stock separated from the adult dairy goats. Ten of the farms routinely vaccinated their goat kids against MAP using the Gudair^®^ MAP vaccine. The farm which sourced the goat kids was included in the cohort of non-vaccinating farms. On all farms, four locations were sampled by collecting samples of settled dust. Two samples were taken in area’s frequented by adult dairy goats i.e., the milking parlour and the waiting area in which goats are temporarily housed prior to entering the milking parlour. In addition, two settled dust samples were taken from the young stock rearing stables. Samples were processed and MAP was detected by IS900 PCR and para-JEM^®^ MAP culturing (TREK Diagnostic Systems, Cleveland, OH, USA) was used to evaluate MAP status of the collected samples as has been described in detail previously [14]. In line with this method we used a combined binomial outcome from both tests per sample (positive/negative).

### 2.2. Animals

#### 2.2.1. Study 1: Goat Kids

Goat kids were sourced in the first quarter of 2015 from a paratuberculosis unsuspected commercial goat farm. The farm history indicated that vaccination against paratuberculosis was never performed, and there were no observed clinical cases of paratuberculosis. The farm was also test negative in bulk milk using a commercial paratuberculosis absorbed ELISA test (see below). In addition, prior to sourcing the goat kids 50 randomly selected dams were tested by milk ELISA, and environmental samples were collected and tested negative by IS900 PCR (see below). Finally, fecal samples were collected from 20 multiparous goats and tested negative by IS900 PCR. Based on the test results there were no indications of the presence of MAP on this farm. 

Prior to arrival of the goat kids the young stock stable in the experimental facility of the Faculty of Veterinary Medicine, Utrecht University (Utrecht, The Netherlands) had been cleaned, disinfected and allowed to dry out according to described protocols [15]. No MAP infected ruminants had been housed in the unit in the year previous to the experiment. 

The farmer selected two batches of 12 twins each (24 animals) born with a 2-week interval thus in total 24 female twins (48 animals) entered the study. During the first 24 hours, the lambs received artificial colostrum (Capracol^®^, Arts Food Products BV, The Netherlands). This colostrum replacer is prepared from bovine colostrum derived from cows from high health herds and gamma-irradiated. On the second day after birth, the goat kids were transported to the experimental facility in a dedicated rapid transporter to minimize transport duration and movement stress. 

Upon arrival, the goat kids were group housed in four units each holding 12 goat kids (6 twins) with ample straw bedding, a heating device for comfort and enrichment in the form of straw bales for climbing. The goat kids had free access to two drinking nipples per group supplying goat kid powder formula on demand as per manufacturer instruction. In addition, the goat kids had free access to grass hay (sourced from a MAP infection unsuspected farm), goat kid concentrate and water. The animals were observed routinely at least twice daily. Animal care taker and research staff access to the stable was restricted to essential dedicated personnel and a strict hygiene and dress protocol was enforced throughout the experiment.

The goat kids were sequentially euthanized in eight sessions with 2-week intervals (starting 14 days prior to vaccination) by lethal intravenous injection with pentobarbital (Euthasol, AST Farma, Oudewater, The Netherlands) and subsequently subjected to post-mortem examination.

#### 2.2.2. Study 2: Adult Goats

In the fall of 2016, 34 female clinically healthy goats selected by the farmer were sourced as dried-off non-lactating animals from three different commercial goat dairy farms in The Netherlands. The farmers considered these goats as healthy herd members, however, decided to cull them based on production history and hence had low motivation to use these goats to breed them for replacement goats. These three farms were not part of the environmental sampling cohort but had a known history of MAP infection and were considered to be MAP endemic properties. Goat kids on these farms were routinely vaccinated against paratuberculosis (see below) as part of farm MAP control management. The average age of the sourced goats was 1.7 years at the time of arrival at the experimental facility of Wageningen Bioveterinary Research (Lelystad, The Netherlands). In total, 25 of the 34 adult goats were born in 2015, 8 were born in 2014 and 1 was born in 2012. The goats were transported to the experimental facility on two separate days, within the same week. The goats were group housed in one unit with free access to artificial dried grass hay and water. The goats received a restricted amount of concentrates on a once daily basis. Goats were housed on ample hemp bedding material and climbing opportunities were available as environmental enrichment. At least twice daily goats were observed by animal care takers and research staff. Following routine procedures of the facility, access was restricted to essential personnel and a strict hygiene and dress protocol was enforced throughout the experiment.

The adult goats were sequentially euthanized starting with 2 goats per week (three days apart) during the first 8 weeks and 1 goat per week for the remainder. The last remaining 2 goats were euthanatized on the same day. All adult goats were euthanized by lethal intravenous injection with pentobarbital (Euthasol, AST Farma, Oudewater, The Netherlands) and subsequently subjected to post-mortem examination.

### 2.3. Ethics

All animal experiments were approved by the animal experiment committees of Utrecht University (goat kid experiment, permit number 2014.II.11.058) and Wageningen Bioveterinary Research (adult goat experiment, permit number AVD401002016580), and were conducted in accordance with the Dutch laws and regulations on animal experimentation.

### 2.4. Post-Mortem Examination

During the post-mortem examination, in addition to general pathological examination the vaccination site was examined including the draining lymph nodes. All goat kids and adult goats were checked for gross lesions indicative of paratuberculosis such as thickening of the mucosa of the distal ileum, mesenteric lymphadenitis, and mesenteric lymphangitis. Samples were taken aseptically from all goats from the distal ileum, distal jejunum, a distal mesenteric lymph node and the ileocecal lymph node for diagnostic MAP-specific IS900 PCR. In addition, samples were taken from tissues with gross lesions for histological examination as well as diagnostic IS900 PCR (see below) to detect paratuberculosis infection.

### 2.5. MAP Infection Status of Goats by IS900 PCR

The details of the IS900 PCR have been published previously [16] with minor modifications as we used tissue samples in the current study. Tissue samples (approximately 3 g per sample) were mechanically homogenized in 10 ml of phosphate buffered saline (PBS). From the homogenate, 200 µL was taken and processed for DNA isolation using the DNeasy blood and tissue kit (Qiagen Benelux, Venlo, The Netherlands) following instructions provided by the manufacturer. The IS900 PCR was run on an Applied Biosystems 7500 Fast Real-time PCR system (Thermo Fisher Scientific, Landsmeer, The Netherlands). The PCR was performed using PerfeCTa Multiplex qPCR supermix UNG (VWR International B.V., Amsterdam, The Netherlands), IS900 F forward primer 5′-CCGCTAATTGAGAGATGCGATT-3′ (400 nM), IS900 Probe 5′- 6FAM – ACCTCCGTAACCGTCATTGTCCAGATCA-BHQ1-3′ (200 nM), IS900 R reverse primer 5′- CCAGACAGGTTGTGCCACAA-3′ (400 nM) and IS900 IPC inhibition control probe 5′-YY-CTGCTGGGTATACGTCGTCTAAGTCCGAATC-BHQ1-3′ (200 nM) in a reaction volume of 20 µL (all primers and probes from Integrated DNA Technologies, Leuven, Belgium). The template DNA consisted of 2 µL of the eluate of the isolated tissue DNA and 1 µL of inhibition control DNA (constructed by designing a non-sense DNA sequence with similar GC content compared to the IS900 amplicon, containing the probe site and flanked by the IS900 primer binding sites). In each PCR run, a negative (nuclease free water) control and a positive (MAP strain B854 genomic DNA) control were included. The reaction conditions were 5′ at 45 °C, 1′ at 95 °C followed by 50 cycles of 10″ at 95 °C; 30″ at 60° C. Data were analysed using the 7500 Fast System SDS software to determine Ct values and check for inhibition to flag potential false negative results. When inhibition was detected samples were rerun at a 1:10 template dilution. For interpretation, Ct values ≤ 36 were labelled positive, Ct values ≥ 40 were considered negative and Ct values between 36 and 40 were classified as inconclusive.

### 2.6. Vaccination

In the goat kid study, one goat kid was randomly selected of the goat kid twins and vaccinated with the commercially available MAP vaccine (Gudair^®^, CZ Veterinaria, Porriño, Spain), the other twin goat kid was not vaccinated and served as non-vaccinated control. The vaccine is an inactivated vaccine against paratuberculosis (Johne’s disease) containing 2.5 mg/mL *M. avium* subsp. *paratuberculosis* 316F strain formulated in a double emulsion with mineral oil as adjuvant. The goat kids were vaccinated as per manufacturer´s instruction with a single 1 ml dose, subcutaneously between the sternum and the right front leg. The goat kids were vaccinated in two batches when goats were approximately 3 weeks of age. 

The goats which were enrolled in the adult goat study had been subcutaneously vaccinated with the commercially available MAP vaccine (Gudair^®^, CZ Veterinaria, Porriño, Spain) in the first month of life with a single 1-ml dose by the dairy goat farm veterinarian. In addition, as per government regulations the adult goats were vaccinated against Q-fever prior to the breeding season with a commercial vaccine (Coxevac^®^, Ceva Santé Animale B.V., Naaldwijk, The Netherlands) according to instructions provided by the manufacturer and described previously [17]. These goats were raised on their respective farm, bred and milked until purchased.

### 2.7. Blood Sampling

In both studies, blood samples were obtained at regular one or two weekly intervals as indicated for a period up to 108 days post vaccination in the goat kids and 112 days post arrival at the experimental facility for the adult goats. Heparinized and serum blood samples were collected in separate Vacutainer tubes (BD, Vianen, The Netherlands) aseptically from the jugular vein of the animals.

### 2.8. Antigen-Specific Antibody Responses

Two commercially available tests were used to measure antigen-specific serum antibodies. Antibodies specific to MAP (goat kids and adult goats) were measured using the ID Screen^®^ Paratuberculosis Indirect test (ID Vet, Montpellier, France). As all bred dairy goats in the Netherlands also receive a yearly mandatory vaccination against Q-fever, antibodies specific to *Coxiella burnetii* (adult goats only) were measured using the IDEXX Q-Fever Ab Test (IDEXX Europe B.V., Hoofddorp, The Netherlands). Both tests were performed as per manufacturer’s instructions.

### 2.9. Antigen-Specific Interferon-Gamma Release Assay

To evaluate vaccination-induced T-cell responses, antigen-specific interferon-gamma (IFN-γ) responses were measured using the whole blood culture Bovigam assay (Thermo Fisher Scientific, Landsmeer, The Netherlands) according to instructions provided by the manufacturer with additional stimulation antigens as modification. In brief, 1.5 mL heparinised whole blood was incubated with bovine and avian tuberculin antigens in a 24-well tissue culture plate for 24 h in a humidified incubator at 37 °C. Nil antigen (PBS) was used to determine spontaneous release of IFN-γ in the blood culture. Subsequently, the supernatant plasma was collected and stored at −20 °C until analysis. In addition, *Coxiella* vaccine antigen as present in the Coxevac^®^ vaccine (CEVA, Naaldwijk, The Netherlands) was used as antigen at a final protein concentration of 6 and 30 µg/mL, and MAP (separately cultured vaccine strain 316F) at a final concentration of 2 × 10^5^ cfu per well based on the manufacturer listed content on the vaccine. The production of caprine IFN-γ was measured using a monoclonal antibody-based sandwich enzyme immunoassay. Results were expressed as S/P ratio calculated as OD450 (antigen stimulated plasma-PBS)/OD450 (positive control plasma-PBS). The positive control plasma was included in the kit.

### 2.10. Statistical Analysis

Data were analysed using GraphPad Prism 7 software (GraphPad Software, San Diego, CA, USA). Depending on the comparisons made the data were log-transformed to obtain normal distribution tested for statistically significant differences with t-tests as indicated. Data for which transformation did not result in obtaining normal distribution were analysed using non-parametric t-tests as indicated. Fisher exact test was used to analyse the contingency tables as indicated. To evaluate the temporal dynamics of vaccine specific immune responses in the goat kid experiment a mixed effect linear model was constructed in R software (version 3.4.0) with random "pair" effect, and vaccination, time and their interaction as well as fixed effects. Residuals of the full model were checked with a normal probability plot. Model reduction was done using Akaike’s Information criterion (AIC) and single term deletion. For the important effect 95% profile log-likelihood confidence intervals were calculated. The model with vaccination × time interaction had the lowest AIC and this interaction was included in the final model. The final model was: lny ~ factor(vac) + factor(time) + factor(vaccination):factor(time) + (1|pair). The level of significance was set at *p* < 0.05.

## 3. Results

### 3.1. MAP in Environmental Settled Dust Samples on Dairy Goat Farms

In total, 79 settled dust samples were collected from the 20 participating farms. One sample from a young stock stable was lost to follow-up. Based on direct IS900 PCR and the culture result 15 out of 79 samples (19%) tested MAP positive. These samples originated from 10 different dairy goat farms. In the milking parlor 7 out of 20 samples (35%) were positive, in the waiting area also 7 out of 20 (35%) were positive. On 4 farms, both the sample from the milking parlor as the waiting area tested positive. In the 39 samples collected from the young stock rearing stables, no MAP was detected. No settled dust samples from the remaining 10 farms tested positive for MAP. In the group of vaccinating farms 6 out 10 farms tested positive, in the group of non-vaccinating farms 4 out of 10 farms tested positive. The farm from which the goat kids were sourced also tested negative in all 4 samples.

### 3.2. MAP Infection Status

No gross lesions were observed in the goat kids, indicative of paratuberculosis and none of the intestinal tissues tested positive with the MAP-specific IS900 PCR. In addition, no PCR inhibition was observed in test negative samples. In all vaccinated goat kids, a local reaction to vaccination was observed (described below). In approximately 25% of the adult goats, a local reaction indicative of vaccination was observed. Whether this was due to the more recent Q-fever vaccination or the MAP vaccination could not be determined. In the adult goats, 2 cases were observed during necropsy in which the ileo-cecal or a mesenteric lymph node was enlarged and had apparent granulomatous macroscopic lesions. Typical granuloma’s were observed by histology in both cases however only in one case a positive MAP diagnosis was made by IS900 PCR. For the remaining 32 goats 3 goats showed a positive IS900 PCR on the randomly sampled non-lesional intestinal tissues, in addition, 3 goats scored an inconclusive PCR response. The 4 goats in which MAP infection was positively diagnosed originated from 2 different farms. Furthermore, in the goats from these 2 farms also 1 PCR inconclusive goat was detected. In one farm, only 1 PCR inconclusive goat was detected based on tissue examination. On sample level the dubious animals (*n* = 3) all had 1 sample dubious and 2 samples negative in IS900 PCR. The 2 positive animals from farm 1 both had 2 samples positive, no inconclusive samples and 1 negative sample. One animal from farm 3 had 1 sample positive and 1 sample inconclusive, 1 sample negative. The other positive animal from farm 3 had 2 samples positive, 1 negative. The results are summarized in Table 1. 

Table 1. Summary of the MAP-specific IS900 PCR results on tissue samples obtained from adult goats at necropsy. Results are presented per farm of origin (1, 2, 3) at animal level. When 1 or more of 3 samples per goat were evaluated as inconclusive or positive the goat is listed here as respectively IS900 inconclusive or IS900 positive (Ct < 36.0). When all samples of a goat were test negative the goat is listed as IS900 negative (Ct > 40.0).

Based on case definitions as proposed by Whittington et al. [18] the adult goats in this study would be classified into 5 categories as described in Table 2 when inconclusive PCR results are considered as positive.

Table 2. Based on case definitions as proposed by Whittington et al. [18] the adult goats in this study would be classified into 5 categories as described in Table 2 when inconclusive PCR results are considered as positive.

### 3.3. Local Vaccine Tissue Responses in Young Goats

The goat kids showed no apparent clinical signs post vaccination apart from palpable swelling at the site of injection. These swellings reached a size up to a diameter of 6 cm across and persisted throughout the experiment up to the final time point. Gross pathology of the vaccination site in all kids indicated an encapsulated area filled with a viscous purulent material. Histological examination typically indicated a chronic granulomatous type lesion with activated lymphocytes, macrophages and limited numbers of neutrophils in connective tissue surrounding necrotic material. 

In addition, there was apparent enlargement of the draining prescapular lymph node in vaccinated goat kids as compared to the hemilateral prescapular lymph node and both the prescapular lymph nodes from the non-vaccinated control sibling. This was observed in all 7 post-vaccination examinations (days 14 through 98).

### 3.4. Vaccination-Induced Immune Responses in Young Goat Kids

**Figure 1:**
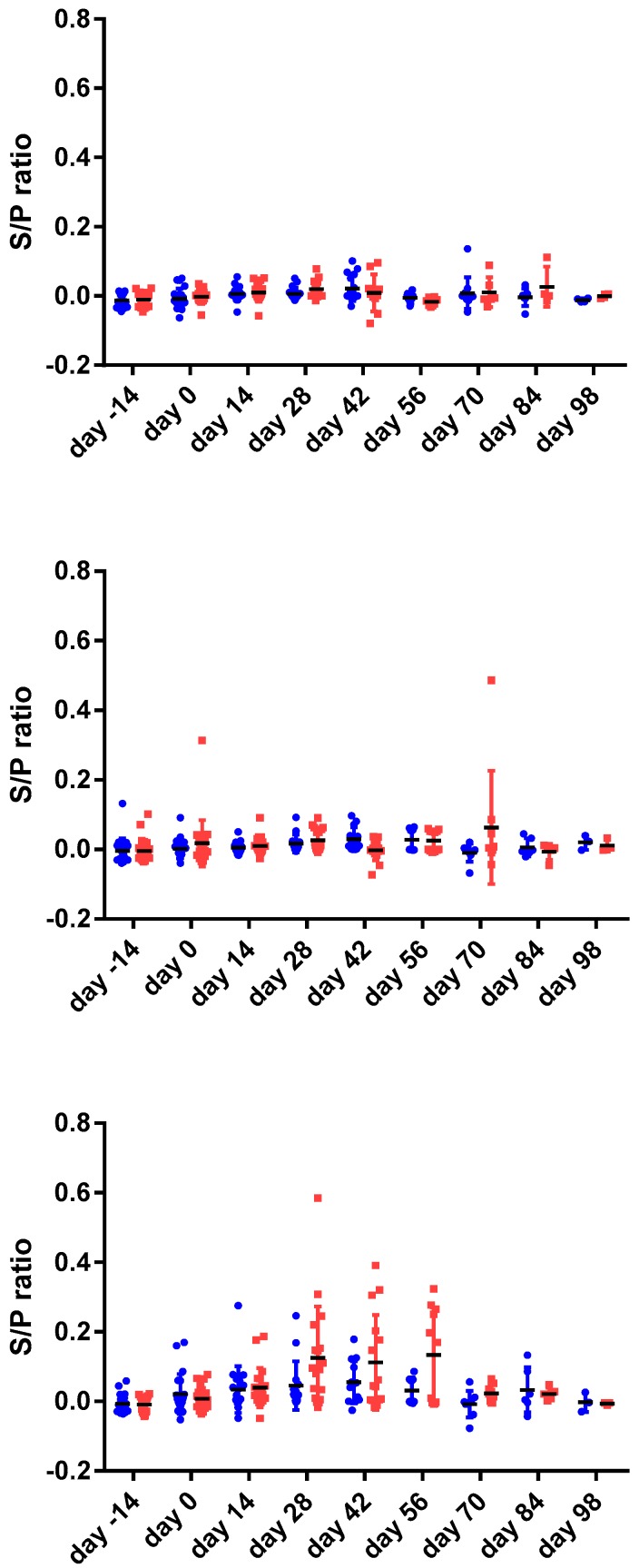
The interferon gamma (IFN-γ) responses (expressed as sample to positive ratio) of 24 goat twins (48 animals) prior to and following vaccination at day 0. IFN-γ responses were measured following 24 h stimulation of heparinized blood with PBS (phosphate buffered saline negative control, top panel), PPDB (*Mycobacterium bovis* tuberculin antigen control, middle panel) and PPDA (*Mycobacterium avium* tuberculin antigen). One of the goat twins was vaccinated (red squares, right side) the other served as non- vaccinated control (blue dots, left side) following acclimatization at day 0. Based on the statistical linear mixed effect model no significant differences were observed between vaccinated animals and non-vaccinated controls.

**Figure 2:**
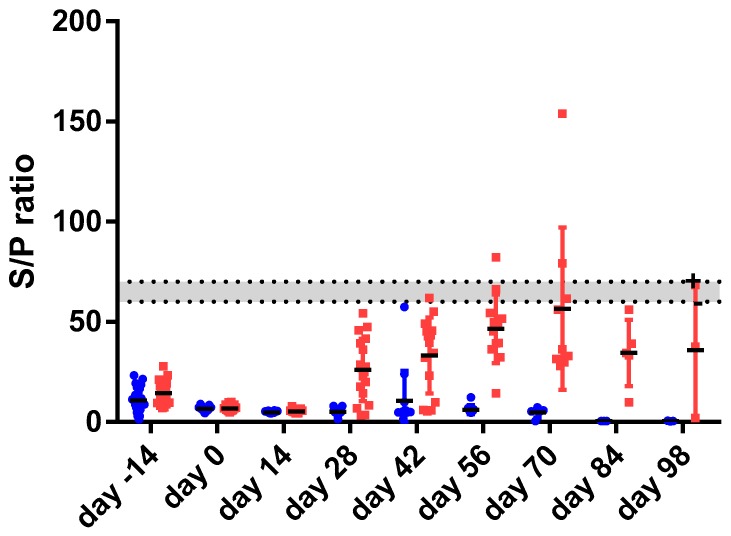
shows the result of the MAP-specific absorbed ELISA for 24 goat twins (48 animals) prior to and following vaccination at day 0. The data is expressed as the sample to positive ratio × 100% calculated as per manufacturer instruction. The horizontal dotted lines represent the test cut-off values for positive (top line, S/P = 70) and negative (bottom line, S/P = 60). The grey area results are classified as dubious. One of the goat twins was vaccinated (red squares, right side) the other served as non-vaccinated control (blue dots, left side) following acclimatization at day 0. Based on the statistical linear mixed effect model significant differences were observed at day 56 and day 70 post- vaccination.

Both, the MAP-specific IFN gamma response (IGRA) (Figure 1) as well as the MAP-specific antibody response measured using commercially available absorbed ELISA tests (Figure 2) were limited and transient in the young goat kids. 

The IGRA (Figure 1) reached an average maximum response between 28–56 days post vaccination. At the end of the study, IFN-γ responses were comparable to those measured in non-vaccinated controls. At no time point were the differences between the non-vaccinated controls and the vaccinated animals significantly different indicating substantial variation in responses in both vaccinated and non-vaccinated groups. 

Similarly, absorbed ELISA antibody responses (Figure 2) in the vaccinated group reached an average maximum response at 56–70 days post vaccination, slightly later as compared to the IGRA and declined after that. According to the absorbed ELISA test cut offs as described by the manufacturer only 4 of the vaccinated goat kids had a formally positive MAP-specific antibody response post vaccination. Based on the LME statistical model there was a statistically significant difference in MAP-specific antibody responses between vaccinates and non-vaccinates at two time points namely at 56 and 70 days post-vaccination (*p* < 0.05). 

### 3.5. MAP-specific Immune Responses in Adult Goats

**Figure 3:**
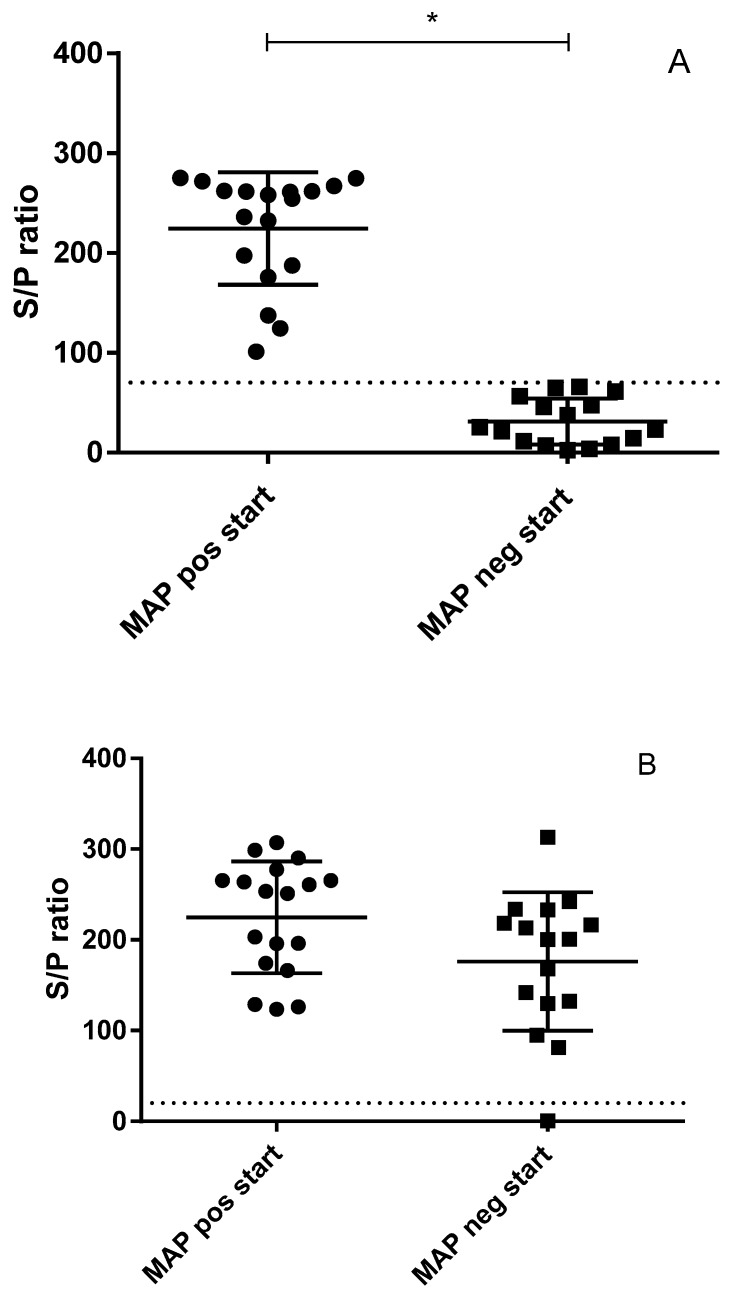
Two commercially available tests were used to measure antigen-specific serum antibodies in 34 adult dairy goats. Panel A: Antibodies specific to MAP were measured using the ID Screen^®^ Paratuberculosis Indirect test (ID Vet, Montpellier, France). Data were grouped according to being positive (dots) or negative (squares) based on the cut-off provided with the test (dotted line at S/P 70) creating a statistically significant difference (p < 0.05) between these two groups as indicated by “*”.. Panel B: As all bred dairy goats in the Netherlands also receive a yearly mandatory vaccination against Q-fever, antibodies specific to *Coxiella burnetii* were measured using the IDEXX Q-Fever Ab Test (IDEXX Europe B.V., Hoofddorp, The Netherlands) (dotted line is the test cut-off at S/P 30). Animals were grouped based on their initial MAP antibody result (initial MAP-positive (dots, left) or initial MAP negative (squares, right)). With respect to their response to the Q-fever vaccination, there was no significant difference between the groups.

As shown in Figure 3, of the 34 goats which were enrolled in the study 18 goats had a positive antibody response in the absorbed ELISA test at the start of the experiment based on the cut-off determined by the manufacturer. Conversely, this also indicated that 16 goats did not have a positive MAP-specific antibody response at 2 years of age despite being vaccinated with a MAP bacterin and being raised in a MAP contaminated environment. The variation of the positive responses in the study population appeared to be extensive and consistent between the first 2 samplings taken a week apart. In comparison, antibody responses to the Q-fever vaccine administered approximately 2 months prior to entering the study indicated that all but 1 goat had a formally positive Q-fever antibody response. The average Coxiella burnetii specific antibody response appeared to be slightly lower in MAP ELISA negative animals as compared to MAP-ELISA-positive animals, however, this difference was not statistically significant.

During the course of being housed in a MAP-free environment in the experimental facility for a period of up to 6 months the MAP-specific antibody responses in the adult goats showed a tendency to decline although this decline was not statistically significant (data not shown).

The initial IFN-γ response to PPDA antigen in the adult goats was highly variable but on average higher as compared to the response to PPDB antigen. This difference was not statistically different. There was no significant difference in IFN-γ response between goats with a positive high antibody response and a negative low antibody response to MAP antigens. There was also no significant difference in IFN-γ response between PCR positive and/or lesional goats and PCR negative goats. Responses to Coxiella antigen were similarly variable.

### 3.6. Relation Between Infection Status and MAP-Specific Immune Responses

As no MAP-positive goat kids were observed no observations were made regarding the relation between MAP-specific immune responses and infection status in goat kids. 

The results of the IGRA test in adult animals indicated that overall MAP-specific IFN-γ responses were variable and appeared not to be related to either vaccination or MAP-specific IS900 PCR infection status (data not shown). If we interpret the data in adult goats based on the criteria for a positive test result in cattle (OD Bovine PPD―Nil antigen ≥0.1 and OD bovine PPD―OD avian PPD ≥ 0.1) we had no adult goats which tested Bovine PPD positive. In total 13 goats had an OD avian PPD ≥ 0.1; from these 13 goats, 1 goat tested positive and 1 goat tested dubious in the IS900 PCR; the remaining 11 goats were IS900 PCR-negative.

Six of the 7 infected goats showed a negative MAP antigen-specific antibody response in the MAP ELISA test. One infected goat had a positive MAP ELISA test. In the 27 non-infected goats 17 goats showed a positive antigen-specific antibody response in the MAP ELISA test. Seven infected goats had a negative MAP ELISA test. The contingency table (Table 3) was analyzed using Fisher’s exact test. The *p*-value was 0.0348. The odds ratio was 0.098 (95% CI 0.008–0.865). The distribution of antibody responders in the infected and non-infected groups was significantly different making it approximately 10 times more likely in this small cohort that a MAP-specific antibody positive vaccinated goat was MAP IS900 PCR negative at tissue level.

Table 3. The relation between IS900 PCR and MAP-specific antibody ELISA test results in the adult goats. The rows represent the numbers of IS900 PCR positive (MAP POS) and IS900 PCR negative (MAP NEG) goats. The columns represent the numbers of MAP-specific antibody ELISA-positive (AB POS) and MAP-specific antibody ELISA negative (AB NEG). The contingency table was analyzed using Fisher’s exact test. The *p*-value was 0.0348. The odds ratio was 0.09 (95% CI 0.008–0.865).

**Figure 4:**
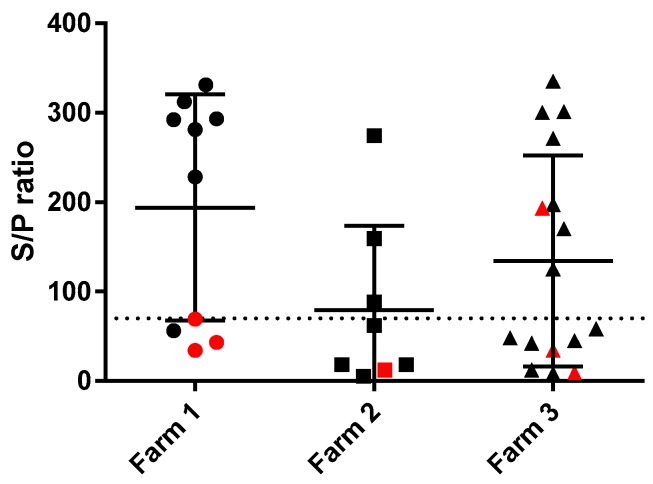
Initial MAP-specific absorbed ELISA antibody responses of adult dairy goats grouped by farm of origin (Farm 1 [circles], Farm 2 [squares], Farm 3 [triangles]). The dotted line indicates the cut-off for the ELISA (S/P 70). The black circles, squares and triangles indicate animals from which tissues showed a negative MAP-specific IS900 PCR result (Ct > 40). The red circles, squares and triangles indicate animals from which tissues showed a MAP-specific IS900 PCR signal (Ct ≤ 40).

The MAP-specific antibody responses of the goats were also compared between the three farms of origin. The MAP-specific antibody responses were not significantly different between goats from different farms although goats from farm 2, which were all born in 2014 and in which only one IS900 PCR inconclusive goat was detected tended to have lower responses as shown in Figure 4. On farm 1 as well as on farm 3 three cases of IS900 positive goats were detected.

The Q-fever specific antibody responses were not significantly different between either MAP PCR positive and negative animals, MAP-specific antibody positive and negative animals, and farms (data not shown). 

## 4. Discussion

The aim of the current study was to first evaluate the early immune response to vaccination in young goat kids sourced from a MAP infection unsuspected non-vaccinated herd and raised in a MAP-free environment. 

Primary vaccination of goat kids in the first month of life with a MAP bacterin vaccine-induced a limited and transient adaptive immune response regarding both MAP-specific antibody responses as well as MAP-specific IFN-γ responses. This is in line with earlier observations [10] in which goats were observed for 9 weeks post vaccination. In our study, we followed the goats for up to 15 weeks post-vaccination. In the additional follow-up period of 6 weeks as compared to the Valheim study, antigen-specific immune responses waned rather than increased. In the Valheim study, it was shown that the increase (although still statistically significant) at 9 weeks leveled off. At the end of the observation period there was no statistically significant difference between the vaccinates and the non-vaccinates for both MAP-specific antibody responses as well as MAP-specific IFN-γ responses. 

Subsequently, we studied the MAP-specific antibody responses as well as MAP-specific IFN-γ immune responses observed in adult goats most of which were born in the same year, similarly vaccinated as kids but raised on farms with documented endemic paratuberculosis. Results indicated that MAP-specific IFN-γ responses did not show a clear relation to MAP infection status in the vaccinated adult goats. It has been documented that in goats MAP vaccination will interfere with routine TB diagnostics (i.e., skin testing with tuberculins) in goats under 20 weeks of age [19]. However there was no interference in vaccinated goats older than 20 months [20] indicating that MAP vaccine-induced T-cell responses wane over time post vaccination.

The MAP-specific antibody responses in the vaccinated adult goats showed a bimodal distribution with clear positive responders and antibody negative animals in near equal numbers. The response to the Q fever vaccine did not show such a distribution indicating that MAP low responders were still able to generate an antigen-specific immune response to the Q fever vaccination. Similar to the IGRA data there was no clear positive correlation of the MAP-specific antibody response with observed infection status in these vaccinated goats. In fact, when relating MAP-specific antibody responders with IS900 tissue PCR results the animals which tested negative in the ELISA were statistically significant more likely to test IS900 PCR positive. 

We hypothesized that the development of adaptive immune responses in goats vaccinated at young age was, at least partially, dependent on the environment they live in. Development of both T-cell and B-cell adaptive immune responses following vaccination of the young goat kids in a MAP-free environment were surprisingly low and short-lived despite a pronounced and sustained inflammatory reaction at the site of injection. In adult goats vaccinated at a similar age and raised in a MAP-endemic environment MAP-specific antibody responses were pronounced in approximately half of the vaccinated goats. MAP-specific IGRA responses in adult goats were variable and not correlated to infection status in those vaccinated goats. Although not statistically significant there also appeared to be a difference between the farms. On farms 1 and 3, more MAP-specific PCR positive animals were detected as compared to farm 2. On farms 1 and 3 also MAP-specific antibody responses tended to be higher in the goats as compared to those from farm 2. The three farms were similar in size and the groups acquired from those farms therefore represented a similar sample size. As the adult goats in these studies were formally recruited for a different study unrelated to paratuberculosis infection and selected by the farmer as being healthy goats in good condition with a less persistent milk production compared to herd mates there was no a priori selection on parameters related to paratuberculosis infection status. As a drawback of this convenience sample and limitation to the data presented, it was not possible to perform a longitudinal study and make a direct comparison between the adult and the goat kid data. 

To study the occurrence of environmental MAP, we focused on bio-aerosols by means of settled dust samples. Bio-aerosols containing lipopolysaccharides of bacterial origin, bacterial DNA and culturable levels of air borne bacteria have been detected in all farm systems studied [21,22]. It has been documented that on cattle farms there is extensive environmental spread of MAP through bio-aerosols [23,24]. Therefore, we hypothesized that environmental exposure to MAP or environmental mycobacteria sharing antigens with MAP could be important for the stimulation of adaptive immune responses following vaccination at young age. The data in the current study indicated that similar to cattle farms, MAP is present in the environment of dairy goat farms with endemic paratuberculosis. More detailed studies regarding spread of MAP through bio-aerosols on dairy goat farms are currently lacking, however, when MAP infectious animals are present on the farm MAP contaminated feces will spread in the environment. Based on our environmental data indicating that in the separate young stock buildings MAP was not detected irrespective of the situation concerning the adult milking goats it can be argued that exposure of young goats to environmental MAP is limited even on MAP endemic farms, similar as described for cattle farms [23]. As such, our study design to raise the goat kids in a controlled MAP-free environment may be comparable to the situation on well managed dairy goat farms with separated young stock rearing and proper management measures. 

In addition, based on our data it is likely that goats vaccinated in the first 6 months of life will primarily get in contact with MAP when they are introduced into the milking herd at approximately 1 year of age where MAP can be readily detected in the environment in half the farms using a limited sampling scheme.

Extensive vaccination field trials have indicated that the vaccine can be effectively used to prevent clinical paratuberculosis, however, infection and transmission still occur, and MAP will likely still be circulating in the farm environment [4]. This is supported by our environmental data showing comparable frequencies of MAP-positive environmental samples when comparing vaccinating and non-vaccinating farms. As we observed in the data obtained from the adult goats, there is a rather clear dichotomy between antibody-positive and -negative adult goats. It may be argued that the serological positive vaccinated goats have been boosted through environmental exposure to MAP. Animals receiving a single vaccination are therefore immunologically primed and potentially through repeated contact with the agent and/or antigens later in life, the adaptive immune responses were enhanced in part of the animals. Whether the fact that approximately half of the goats show this enhancement depends on (level of) contact with relevant mycobacterial antigen pre- or post-vaccination or the way animals were primed by the vaccine is currently unknown. We decided to vaccinate the animals as early as indicated by the manufacturer (2–3 weeks of age) as this is the most common practice in Dutch dairy goat farms, however, the vaccine can be used in adult goats as well. Corpa et al. [12] studied the effect of age at which goat kids were vaccinated and observed that between vaccinating at 15 days of age and 5 months of age, there was no significant difference although IFN-γ responses tended to disappear earlier in animals vaccinated at 15 days old. The antibody response observed by Corpa et al. was always higher and more persistent in animals vaccinated at 5 months as compared to 15 days of age at vaccination—possibly related to maturity of the immune system at the different ages [12]. Our data are in line with the data of the young vaccinated groups in the Corpa study. Storset et al. [11] evaluated IFN-γ responses in goats from farms with or without MAP vaccination and different infection status. They reported that animals from vaccinated herds with paratuberculosis showed higher IFN-γ responses as compared to animals from the vaccinated only herds. In both these groups the IFN-γ responses were correlated to age with higher responses in younger animals. The latter observation is in line with our current observations in the vaccinated goat kids. Our IFN-γ responses in the adult goats are less clear as compared to the observations made by Storset et al., however, we do not have a clear explanation for the difference [11].

When applied in a herd with endemic MAP infection, the vaccine will in at least a number of cases be applied as a post-exposure vaccine i.e., animals will be exposed to MAP at or prior to application of the vaccine. The later the vaccine is applied the greater the chance that animals encounter MAP and related environmental mycobacteria. This exposure may affect vaccine efficacy in a positive or negative fashion depending on route of exposure and viability of the environmental mycobacteria as has been shown for BCG vaccination in murine models and cattle [25,26]. It has been shown that in experimental setting post-exposure vaccination strategies against MAP can be efficacious [27,28]. 

A secondary question was whether the observed immune responses also had implications with regard to infection status or opportunities for immune diagnostic tests in vaccinated populations of dairy goats. Surprisingly, we determined that vaccinated goats in which we were unable to detect infection at necropsy had statistically significant higher antibody responses as compared to vaccinated infected individuals. In addition, IFN-γ responses did not differ significantly between these groups. The results indicated that in these vaccinated goats the higher post vaccination antibody response was associated with lower probability of being PCR positive. During natural infection without vaccination higher antibody responses during later stages of disease are commonly associated with progressive infection, however, these observations to some degree depend on the methods used to evaluate the antibody response [6,29]. In addition, it has been shown that MAP subunit vaccine-induced antibody responses better associate with protection as compared to T-cell responses in cattle [27,28]. The current studies support observations that protection from MAP infection is associated with vaccine-induced antibody responses. In light of the debate regarding the nature of the immunological correlates of vaccine-induced protection these data again highlight the need to include detailed studies on the role of antibodies in the protective immune responses against MAP infection and intracellular bacterial infections in general [30]. 

Mathematical modelling studies of the effects of using an imperfect vaccine (infection and transmission not completely blocked) for control of MAP indicated that it is important to use such vaccines as part of a comprehensive control program [31]. With respect to the consequences for immune diagnostic procedures in vaccinated animals, we presented observations regarding a specific 6 month time-frame starting with animals which were on average 1.7 years of age. As the study with the goat kids observed animals up to 6 months of age there is approximately a 1 year gap in between for which currently no information is available. We selected these periods as a representative time at which farmers often select animals for culling based on development, productive performance and health. In the observation period of the adult goats we noted that the IGRA data consistently showed no differences between IS900 PCR positive and negative vaccinated animals. In addition, the MAP-specific antibody responses were inversely correlated with MAP IS900 PCR positive individuals. We did not determine shedding of MAP in feces in this study and therefore cannot qualify infectivity of the PCR positives. However, selection of ELISA-positive adult vaccinated goats as a test-and-cull measure to remove infected/infectious individuals should be discouraged based on the fact that our current data indicates an inverse relation. As a diagnostic alternative fecal culture or PCR should currently be the test of choice for a test-and-cull strategy in vaccinated goats although this is likely too expensive for extensive routine screening in dairy goat husbandry. Additional management strategies such as preventing exposure to high doses of MAP at an early age are important in control as it has been shown that in calves later exposure with lower doses limits early infectivity and delays progression from latency [32]. Data from the environmental sampling indicated that MAP circulates in the environment of dairy goat farms similarly to dairy cattle farms and that spatially separated housing of young goat kids efficiently limits exposure to MAP early in life. In conjunction with the vaccine effects, decreasing shedding and incidence of clinical cases such management systems will enable better control of caprine paratuberculosis. Similar to Roy et al. [33], no false positive bovine PPD reactors in the Bovigam test in the TB-free adult goats a year or longer after MAP vaccination were observed, which is important when considering the monitoring of TB infections.

## 5. Conclusions

Results from our studies indicated that initial adaptive immune responses to vaccination are limited in a MAP-free environment. In addition, we showed that adult MAP antibody-positive vaccinated goats raised in an endemic environment are less likely to be MAP-PCR-positive as compared to antibody negative herd mates. We conclude that test-and-cull strategies for paratuberculosis in a MAP-vaccinated TB free herd are currently not feasible using available immune diagnostic tests.

## Figures and Tables

**Table 1:** *Mycobacterium avium* subsp. *paratuberculosis* (MAP)-specific IS900 PCR results on tissue samples obtained from adult goats.

Farm	1	2	3	Total
IS900 negative (Ct > 40.0)	7	7	13	27
IS900 inconclusive	1	1	1	3
IS900 positive (Ct < 36.0)	2	0	2	4
Total	10	8	16	34

**Table 2:** *Mycobacterium avium* subsp. *paratuberculosis* (MAP) case definition of goats.

Category	Sub-category	*n*
Exposed to MAP		34
Not infected by MAP		27
Infected with MAP		7
Histological lesions	Diseased-subclinical	1
No lesions	Subclinical infection	6

**Table 3:** Relation between ***Mycobacterium avium* subsp. *paratuberculosis*** (MAP) infection status and antibody response.

*MAP Infection Status*	AB POS	AB NEG	Total
MAP POS	1	6	7
MAP NEG	17	10	27
Total	18	16	34
